# Power Spectral Density Analysis for Optimizing SERS Structures

**DOI:** 10.3390/s22020593

**Published:** 2022-01-13

**Authors:** Ekaterina Babich, Sergey Scherbak, Ekaterina Lubyankina, Valentina Zhurikhina, Andrey Lipovskii

**Affiliations:** 1 Laboratory of Multifunctional Glassy Materials, World-Class Research Center “Advanced Digital Technologies”, Peter the Great St. Petersburg Polytechnic University, Polytechnicheskaya 29, 195251 St. Petersburg, Russia; babich.katherina@gmail.com (E.B.); sergeygtn@yandex.ru (S.S.); zhurihina_vv@spbstu.ru (V.Z.); 2 Laboratory of Nanophotonics, Alferov University, Khlopina 8/3, 194021 St. Petersburg, Russia; 3 Laboratory of Optics of Heterogeneous Structures and Optical Materials, Alferov University, Khlopina 8/3, 194021 St. Petersburg, Russia; katylubyankina@gmail.com; 4 Scientific Educational Center “Physics and Technology of Heterogeneous Materials and Nanoheterostructures”, Institute of Physics and Mechanics, Peter the Great St. Petersburg Polytechnic University, Polytechnicheskaya 29, 195251 St. Petersburg, Russia

**Keywords:** power spectral density, metal nanostructures, surface enhanced Raman scattering

## Abstract

The problem of optimizing the topography of metal structures allowing Surface Enhanced Raman Scattering (SERS) sensing is considered. We developed a model, which randomly distributes hemispheroidal particles over a given area of the glass substrate and estimates SERS capabilities of the obtained structures. We applied Power Spectral Density (PSD) analysis to modeled structures and to atomic force microscope images widely used in SERS metal island films and metal dendrites. The comparison of measured and calculated SERS signals from differing characteristics structures with the results of PSD analysis of these structures has shown that this approach allows simple identification and choosing a structure topography, which is capable of providing the maximal enhancement of Raman signal within a given set of structures of the same type placed on the substrate.

## 1. Introduction

By now, Surface Enhanced Raman Scattering (SERS) has become a widespread method of highly sensitive structural and compositional analysis of objects related to biology, chemistry, condensed matter physics, etc. Presently it is considered as a platform for sensing of chemical and biological impurities for ecology [1], pharmacology [2], biology [3] and chemistry [4]. Raman signal enhancement is due to an increase in local electric field of both incident and scattered light wave near discontinuities of metal structures used as SERS substrates. Plasmonic resonant properties of such substrates and nonlinearity of Raman scattering provide 5–8 orders of magnitude enhancement of Raman signal by metal nanostructures [5,6,7,8], and the enhancement up to 10^10^–10^11^ is predicted [9]. A variety of metal structures providing the enhancement has been demonstrated by now, from rough surfaces [10,11] and metal island films [12,13] to flower-like structures [14], dendrites [15,16], structures formed using organic templates [17], microsphere lithography [18], e-beam lithography [19] and many others. Structures formed using “bottom-up” approach are in the common use because of the simplicity of fabrication. However, such structures allow mainly statistical description, and establishing clear relation of their topography and Raman scattering enhancement is a challenging problem. Essential efforts were directed to design a general approach to optimization of SERS substrates [20,21,22], however this problem stays under consideration. Principally, higher surface concentration of “hot spots” provides higher enhancement [23], and this was demonstrated via deducing distribution of distances between adjacent approximately hemispherical metal islands in SEM images of SERS substrates [24]. Nevertheless, this is neither a universal recipe nor universal criteria, for SERS substrates are complex, multiply connected metallic micro- and nanostructures containing elements, the size and shape of which can vary significantly. It is intuitively clear that the enhancement of SERS by a substrate is somehow related to its roughness; however, as far as we know, this relationship has not yet been quantified. In this paper we demonstrate that second order statistics, namely Power Spectral Density (PSD) analysis developed for signal processing, spectra, noise and other random processes [25,26,27,28], and which is in a wide use for surface roughness characterization [29,30,31,32,33,34,35], establishes a direct correlation of SERS abilities of metal structures and their topography. The use of PSD calculations, available, for example, using MatLab^®^ or Gwyddion^®^ software, provides a numerical criterion for evaluating the performance, thus optimizing the configuration of several specific SERS substrate types.

In the present study, we developed a model, which randomly distributes hemispheroidal particles with given average parameters (radius, height, dispersion) over a given area. We analyzed PSD functions of the modeled structures with different parameters. Also, we applied PSD analysis to atomic force microscope (AFM) and optical profilometer images of a set of differently fabricated on the glass surface metal island films and metal dendrites. As a result, we revealed that the inflection point of the PSD function plotted in a log-log scale relates to a lateral correlation length of the substrate surface and directly correlates with Raman signal enhancement.

## 2. Materials and Methods

### 2.1. Power Spectral Density Analysis

The one-dimensional PSD function of the structure surface profiles, e.g., along the chosen line of AFM scan, represents as follows [36]:

(1) $$PSD(\omega )=\frac{2\pi }{M_{x}M_{y}d}{\sum_{j=0}^{M_{y}-1}\left|{\hat{P}}_{j}(\omega )\right|}^{2},$$

where ω is the spatial frequency, ${\hat{P}}_{j}(\omega )$ is the Fourier coefficient of the *j*-th profile defined as:

(2) $${\hat{P}}_{j}(\omega )=\frac{d}{2\pi }\sum_{n=0}^{M_{x}-1}z_{nj}\exp(-i\omega nd),$$

*M_y_* and *M_x_* are numbers of profiles and points in a profile, respectively, *d* is a pixel dimension along the line, and $z_{nj}$ is the value of height at the *n*-th point in the *j*-th profile. 

**Figure 1:**
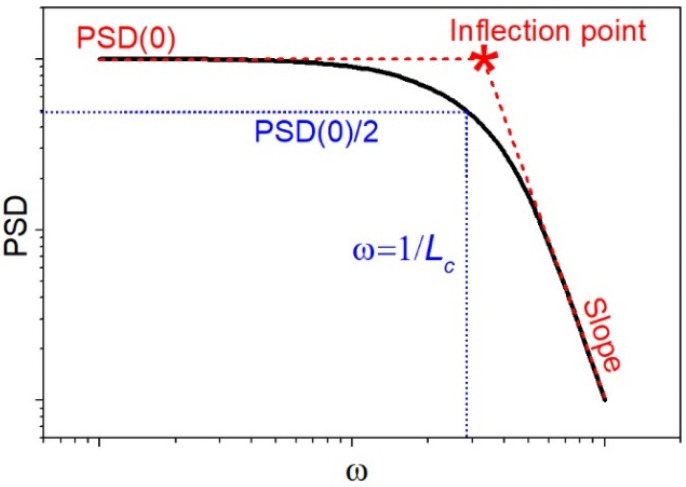
Schematic visualization of a typical PSD function of a randomized surface in a log-log scale. Low-frequency level (*PSD*(0)), high-frequency sloped region and their infection point are denoted, as well as frequency corresponding to correlation length, *L_c_*.

In literature, there are different approaches to analyze a PSD function, which depend on structures and phenomena under consideration. A widely used one is to approximate the function with a piecewise function. Particularly, Shifted-Gaussian function decently approximates the low-frequency range of the PSD, and so-called ABC and fractal models are applicable for the approximation of the high-frequency range [29,30,37]. However, complex surface topography requires a combination of these models as a fitting function for the whole spectral range [38]. This increases the number of fitting parameters up to eight and makes the fitting function, as well as the fitting parameters, not uniquely defined. Another approach is to qualitatively and quantitively characterize the PSD function plotted in a log-log scale: determine the low-frequency region where the PSD is constant (*PSD*(0)) and the high-frequency region where the PSD represents a straight sloped line, determine the point at which the function curves down (spatial frequency corresponding to the inflection point) and the slope of the function in the high-frequency region [39,40]. Note, the substrate surface is considered uncorrelated in the “flat” region of the PSD function and correlated at the frequencies higher the inflection point. In Figure 1 we show a typical PSD function of a randomized surface with the definition of all the parameters to be discussed. From this simple analysis, one can determine the surface roughness exponent (Hurst exponent) via the slope, the variance (root mean square roughness) via the area under the curve, and the lateral correlation length, *L_c_*. There are several definitions of the lateral correlation length, e.g., as the spectral position (inverse abscissa, *ω*) of the inflection point [39] or as the inverse abscissa of the point corresponding to the 1/e level of *PSD*(0) [40]. However, the approach to the *L_c_* definition does not essentially affect the *L_c_* dependence on the surface parameters. In this study we used the following definition [41]:

(3) $$PSD\left(\frac{1}{L_{c}}\right)=\frac{PSD(0)}{2}$$

Concerning SERS, height distribution of the particles (surface roughness) is less important than their lateral distribution, since the region of maximal enhancement of the electric field corresponds to the gap between two closely placed particles, i.e., “hot spot”, for light incidence normal to the surface. Therefore, of all the PSD parameters described above, the lateral correlation length is of the main interest in the study of SERS applicability of the structures.

To reveal the correlations between *L_c_* and SERS, we performed a topographical and electrodynamic modeling of a substrate represented as an ensemble of silver hemispheroidal nanoparticles on a glass surface and verified the results with the experimental data.

### 2.2. Developed Model

To simulate the surface topography of the structure formed by *N* hemispheroidal nanoparticles placed on a flat surface we generated a $\left(X_{n}^{0},Y_{n}^{0},R_{n}^{}\right)$ array, where $X_{n}^{0}$ and $Y_{n}^{0}$ are the coordinates of the particles’ centers, *R_n_*—the particles’ radius, using the algorithm presented in our previous work [24]. First, we set a log-normal distribution of the lateral sizes (*R_n_*) of the nanoparticles, $R_{n}\propto \ln N(\bar{R},\sigma^{2})$, with a given average radius, $\bar{R}$, and dispersion, σ. Second, we set a random position, $\left(X_{n}^{0},Y_{n}^{0}\right)$ (*n* varies from 1 to *N*), in a 1 × 1 µm^2^ (or any given) area for each nanoparticle, avoiding the particles overlapping. The latter means the relation ${\left(X_{n}^{0}-X_{k}^{0}\right)}^{2}+{\left(Y_{n}^{0}-Y_{k}^{0}\right)}^{2}\geq {\left(R_{n}+R_{k}\right)}^{2}$ must hold for any n≠k. Next, to obtain the actual topography of the structure generated, we constructed a grid $\left(x_{i}^{},y_{j}^{}\right)$ over the area, where *i* ranges from 1 to *M_x_* and *j*—from 1 to *M_y_*. The grid is uniform, i.e., $x_{i+1}^{}-x_{i}^{}=\Delta x$ and $y_{j+1}^{}-y_{j}^{}=\Delta y$, and Δx=d in terms of notations introduced in Equation (1) and Equation (2). We attributed a height *z_ij_* to each point of the grid as $z_{ij}=0$, if the point $\left(x_{i}^{},y_{j}^{}\right)$ is “outside” any of the particles; and

(4) $$z_{ij}=\beta \sqrt{R_{n}{}^{2}-{\left(x_{i}^{}-X_{n}^{0}\right)}^{2}-{\left(y_{j}^{}-Y_{n}^{0}\right)}^{2}},$$

if the point $\left(x_{i}^{},y_{j}^{}\right)$ is “inside” the *n*-th particle (*n* ranged from 1 to *N)*. In Equation (4) the aspect ratio *β* = 1 for hemispherical particles, and *β* = *h/R_n_* for hemispheroidal ones (*h* is the particles’ height). Thus, we constructed an array $\left(x_{i}^{},y_{j}^{},z_{ij}\right)$ that describes the topography of the surface consisting of randomly distributed hemispherical/hemispheroidal particles. The arrays of this kind are equivalent to experimentally obtained AFM-profilograms.

The 1D PSD function based on the generated array was calculated and the correlation length was evaluated using Equations (1), (2) and (3), respectively. To estimate the dependence of *L_c_* on the surface topography parameters we analyzed a set of arrays differing in the number, average radius, and height of nanoparticles. 

To reveal the correlation between *L_c_* and Raman enhancement, we performed modeling of the electrodynamic characteristics of the same ensembles of nanoparticles. First, using finite elements method in COMSOL Multiphysics^®^, we calculated the dependence of the average electric field in a gap between two particles of different sizes on the distance between them (interparticle gap), *g*. We considered silver hemispheroidal nanoparticles on a substrate with the refractive index of 1.5 (glass) excited by normally incident light at a wavelength of 633 nm, which is relatively far from the plasmon resonance wavelength of silver nanoparticles (~420 nm [13]). This allowed us to neglect optical resonant effects, in particular, the dependence of the resonant wavelength on the *g* and focus on the morphology influence on the optical performance of the structure. Secondly, we calculated gaps between all nanoparticles in a given ensemble and estimated the number of “hot spots”, *N*_2_, i.e., the number of particles’ pairs with a relatively small interparticle gap, which presumably give the overwhelming contribution to the SERS signal. We followed ref. [24] and considered as a “hot spot” a pair of nanoparticles with the gap less than 0.5 of the average radius of these pair of nanoparticles, $R_{av}=(R_{1}+R_{2})/2$. 

Finally, to estimate Raman signal (in arbitrary units), the calculated electric field in the 4th power was summed up over all “hot spots” [42,43]:

(5) $$G\sim \sum_{i=1}^{N_{2}^{}}\langle {\left|E_{i}(g_{i})\right|}^{4}\rangle ,$$

where <*E_i_*>—the average electric field of *i*-th “hot spot”, which depends on its gap *g_i_*, *N*_2_—the total number of “hot spots”. Note, here we did not take into account Raman enhancement by single nanoparticles and by “hot spots” of higher orders (triplets, quaternaries, etc.). This is because the enhancement by a single nanoparticle in the non-resonant regime is relatively low and the number of “hot spots” of higher orders is also almost negligible for reasonable fill factors, *FF*, that is the percentage of the substrate area occupied by nanoparticles to the total substrate area:

(6) $$FF=\frac{\sum_{i=1}^{N}\pi R_{i}{}^{2}}{1\mu m^{2}}\cdot 100\%\approx \frac{N\pi {\bar{R}}^{2}}{1\mu m^{2}}\cdot 100\%$$

Further, we compared the obtained results of topographic and electrodynamic modeling with experimental data for silver nanoisland films, the surface topography of which coincides with the modeled one, and for silver dendrites with topography essentially different from the developed model.

### 2.3. Experimental

Both types of silver nanostructures on the glass surface, nanoisland films and dendrites, were fabricated, characterized and tested in SERS. The difference between structures was that nanoisland films were ensembles of hemispheres/hemispheroids randomly distributed on the substrate surface, while dendrites were nanorods stuck out of the substrate. Both structures were formed by self-assembly of silver atoms in glass matrix.

It is known that one can introduce silver ions into a glass network by silver-to-sodium ion exchange [44]. The replacement of sodium ions in glass by silver ones occurs when the glass is immersed in a silver-containing molten salt at elevated temperature. We used soda-lime glass slides containing 14.3 wt.% of Na_2_O, 6.4 wt.% of CaO, 4.3 wt.% of MgO, 1.2 wt.% of K_2_O and 1.2 wt.% of Al_2_O_3_ (Agar Scientific Ltd., Essex, UK), and mixture of silver and sodium nitrates (LenReactiv, St. Petersburg, Russia) with AgNO_3_ molar concentration of 2.7%. The glass slides were cleaned in acetone:isopropanol solution and immersed for 20 min into the molten salt bath heated up to 325 °C. As the result, silver ions penetrated into the glass to the depth of about 7 μm (zero silver concentration level) and the maximum silver oxide concentration at the glass surface was about 10 mol.% [45].

To fabricate silver nanoisland films (SNF) we annealed silver-containing glasses in a hydrogen atmosphere. Hydrogen, diffusing into the subsurface glass layer, reduces silver ions to atoms, and further out-diffusion and clustering of silver atoms results in the formation of hemispherical/hemispheroidal nanoparticles (nanoislands) on the glass surface [46]. We have demonstrated in our previous work [24] that the duration of the annealing at a fixed temperature governs the spatial and size distribution of the formed nanoislands, and, consequently, Raman enhancement. Repeating these results, we annealed the glasses for 5, 10, 15 and 20 min at 250 °C.

Silver dendrites were fabricated via electrolysis of silver-containing glass [16]. We deposited 50 nm-thick aluminum electrodes on the opposite sides of the glass slides, heated the slides up to 250 °C and applied 100–600 V DC voltage. Silver ions present in the glass drift towards the cathode under the voltage applied. Approaching the cathodic surface, ions are reduced by electrons to silver atoms, and cluster into dendritic forms. Dendrites grow along the electric field lines, from the cathode towards anode, and we assume that with the increase of the charge passed through the glass, the length and fractality of the dendrites increase. By varying the applied voltage and electrolysis duration, we prepared four samples differing in the charge passed: 0.08, 0.21, 0.37 and 0.59 C. Since the dendrites were formed in the subsurface glass layer, after the electrolysis we etched off the aluminum electrodes in 50% water solution of NaOH (1 h-etching) and removed the glass surface layer in NH_4_F/HF solution, 5 µL HF: 5 g NH_4_F: 40 g H_2_O (10 min-etching). 

The surface topography of the fabricated nanostructures was characterized by atomic force microscopy (NTEGRA Spectra, NT-MDT, Russia; DIMENSION-ICON, Bruker, Billerica, MA, USA) and optical profilometry (NewView 6000, Zygo, Middlefield, CT, USA). We used fine AFM probe with a tip of 2 nm in radius (ScanAsyst-Air, Bruker, Billerica, MA, USA) to characterize SNFs, taking into account that the typical size of the nanoislands was about 10 nm, and we used the AFM tip with a 10 nm curvature radius (Etalon, NT-MDT, Moscow, Russia) for dendrites, whose size was about 1 µm. Additionally, considering fractal nature of the dendrites and their nonuniform spatial distribution, we obtained large-scale images of the substrates with the dendrites using optical profilometer equipped with 50x objective. The processing of the AFM and optical profilometer images, including calculation of 1D PSD function based on profiles along the scanning line, was made using Gwyddion^®^ software. The PSD functions based on AFM and optical profilometer images of the same substrate were superimposed on each other to provide accurate input of higher and lower spatial frequencies and the resulting curve was analyzed.

To study the impact of surface topography on Raman enhancement provided by the nanostructures, we performed SERS measurements using low-NA objective, averaging signal over an ensemble of nanostructures and eliminating the influence of the possible signal spikes. Non-resonant excitation allowed us eliminating the impact of optical resonances in nanostructures on the enhancement. We used confocal Raman microscope with 10x/0.3 objective and 632.8 nm excitation laser (LabRAM HR800, Horiba, Japan) to study SNF, and 532 nm laser (Alpha 300R, Witec, Germany) to study dendrites. To avoid resonant Raman scattering we used a test analyte that does not absorb light in the visible spectral range, 1,2-di(4-pyridyl)ethylene (so-called BPE) [47]. The droplets of BPE water solution were dried on the substrates’ surface, the molecular coverage being ~10^−11^–10^−10^ mol/mm^2^. The Raman spectra acquisition time and laser power density were 30 s and 7 mW, respectively, in the case of SNF study, and 1 s and 0.7 mW for dendrites study. Note that it is hardly possible to directly compare SERS signal provided by different types of nanostructures because different microscopes and excitation wavelengths were used.

## 3. Results and Discussion

### 3.1. Experimental

#### 3.1.1. Electrodynamic Modeling

**Figure 2:**
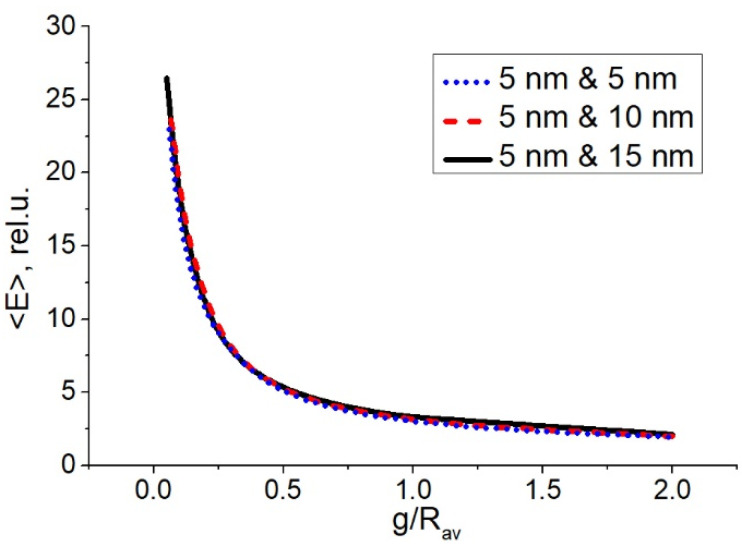
Dependence of the average electric field, <*E*>, in a gap between two particles on the interparticle gap, *g*, normalized by the average radius of the particles, *R_av_*. Radius of particles in pairs: 5 nm and 5 nm, 5 nm and 10 nm, 5 nm and 15 nm.

Using the developed model (see Section 2.2) we calculated the dependence of the average electric field, <*E*>, on the interparticle gap, *g*, for pairs of particles with different radius: 5 nm (1st particle) and 5 nm (2nd particle), 5 nm and 10 nm, 5 nm and 15 nm. The results of the calculations are presented in Figure 2. We normalized *g* on the average radius of particles in the pair, *R_av_*. The incident electric field magnitude was unit; therefore, the calculated electric field represents just field enhancement by a pair. Note that we considered only the incident wave polarized along the interparticle axis. For an arbitrary angle *φ* between the light polarization and the interparticle axis, the resulting electric field will scale at a factor of |cosφ|. Averaging the electric field over all the interparticle directions in a random nanoisland film results in a constant factor 2/π, which is out of interest and is omitted further. 

The dependences in Figure 2 show that the field in the gap, expectedly, is higher for smaller gaps. Particularly, field enhancement reaches values of the order of 15–20 and more for gaps less than 0.1 *R_av_* that is relatively high considering the non-resonant excitation. One can see that the dependencies in Figure 2 are almost identical, i.e., the field enhancement by “hot spots” is the same for the same ratio *g/R_av_*, and does not depend on the particles’ sizes. Thus, we are able to use the universal dependence presented in Figure 2 to estimate Raman enhancement over all “hot spots” in the ensemble of silver nanoparticles. However, this is valid, while the particles are much smaller than the wavelength of the light, since for larger particles wave retardation can affect the field enhancement. Also, this is valid, while the excitation is non-resonant, i.e., the dependence of the plasmon resonance wavelength on the distance between particles in a pair is out of the frames of the model.

#### 3.1.2. Modeling of PSD and Raman Enhancement

**Figure 3:**
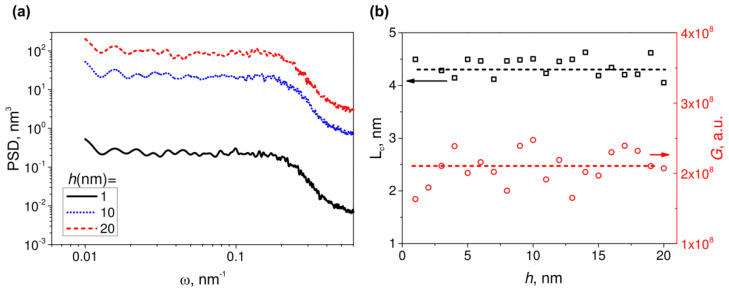
(**a**) Modeled PSD functions for ensembles of 1000 hemispheroidal particles with the average radius 10 nm and the radius dispersion 2 nm, randomly distributed over an area of 1 × 1 μm^2^; height of the particles in each ensemble is 1, 10 and 20 nm. (**b**) Dependencies of the correlation length (left axis, black squares) and Raman signal (right axis, red dots) on the height of the particles. The flat lines are guides for eyes.

To reveal the correlation between the shape of the PSD function, Raman enhancement and the parameters of an ensemble of hemispheroidal nanoparticles, we modeled sets of different ensembles. First, we investigated the influence of the nanoparticles’ height on the PSD. We considered the ensembles with the same number (*N* = 1000), the average radius ($\bar{R}$ = 10 nm) and the radius dispersion (*σ* = 2 nm) of nanoparticles but different heights. Note, the height of the particles changed from ensemble to ensemble, while all the particles in the ensemble were of the same height. In Figure 3a we present obtained PSD functions calculated for three different heights *h*, and Figure 3b shows the correlation length, *L_c_*, calculated from the PSD functions of modeled structures and Raman signal in an arbitrary scale, *G*, versus the height of the structures.

In Figure 3a one can see typical PSD functions of randomized surface with low-frequency flat region and high-frequency sloped region. With increasing the height of the particles, the inflection point and the slope of the curves stay relatively the same, whereas the magnitude of the PSD function drastically increases. There is a slight bending of curves at the higher frequencies, which is caused by numerical errors and can be “straightened” via fining of a profile discretization, i.e., increasing *M_x_* and *M_y_*. In Figure 3b one can see that, as expected, the correlation length and the Raman signal stay approximately constant with varying of the nanoparticles’ height. Indeed, height distribution, though important for PSD analysis in general, is almost irrelevant for SERS in particular, since “hot spots” are located at a lateral plane near the substrate surface and effect only the components of the electric field, which are parallel to the substrate. Note that some fluctuation in plots is because of the stochastic nature of the modeling.

**Figure 4:**
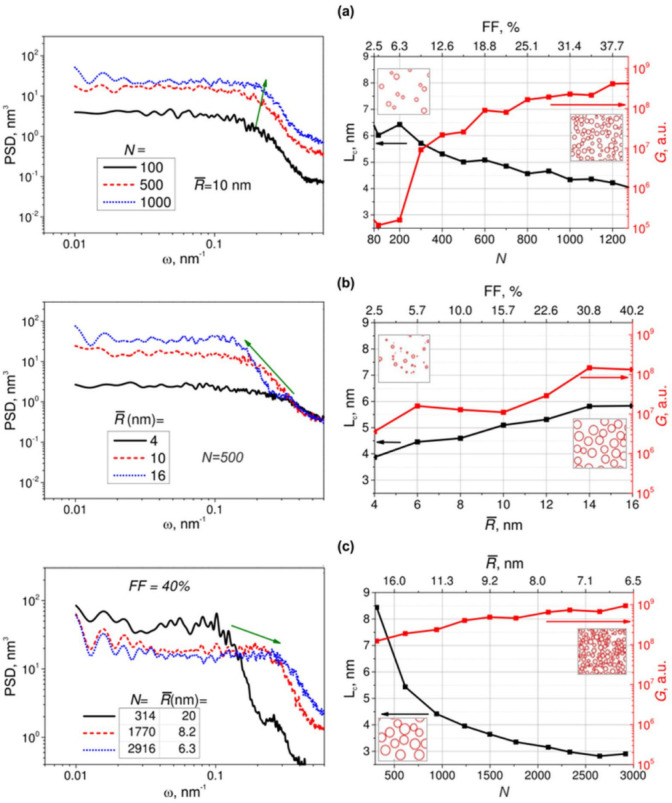
Modeled PSD functions (left column) and dependencies of the correlation length (left axis, black curve) and Raman signal (right axis, red curve) (right column) for the ensembles of particles with: (**a**) variable number and the same average radius of particles, $\bar{R}$ = 10 nm (insets: simulated random spatial arrangements of *N* = 100 and *N* = 1000 particles); (**b**) variable average radius and the same number of particles, *N* = 500 (insets: simulated random spatial arrangements for the ensembles with $\bar{R}$ = 4 nm and 16 nm); (**c**) variable number and average radius of particles, but with the same fill factor, 40% (insets: simulated random spatial arrangement of *N* = 314 particles with $\bar{R}$ = 20 nm and *N* = 2916 particles with $\bar{R}$ = 6.3 nm). In all the computations, the surface area is 1 × 1 μm^2^, the nanoparticles have log-normal radius distributions, and the radius dispersion is 2 nm.

Next, we studied the influence of the spatial distribution of nanoparticles on the shape of PSD function and Raman signal. We considered the ensembles with the nanoparticles of the same height (*h* = 10 nm) and radius dispersion (*σ* = 2 nm) but varied the average radius and the number of nanoparticles. We carried out three series of computation: with fixed $\bar{R}$ and variable *N*; with fixed *N* and variable $\bar{R}$; and with both *N* and $\bar{R}$ being variable, but keeping the fill factor, *FF* (see Equation (6)), of the film constant. In Figure 4 we present the results of this modeling and examples of spatial distributions of particles obtained in these simulations (only limiting cases).

In Figure 4a we observe that with the increase of number of particles in the ensemble, the PSD function grows in magnitude and the inflection point shifts to the high-frequency region, the shape of the function being approximately the same. The magnitude growth, as it is shown in Figure 3a, is related to the increase of the metal volume in the structure and, respectively, the average height of the whole structure with the increase of number of particles. The shift of the inflection point indicates a decrease of the correlation length that is expected for ensembles with densely packed particles (highly periodic structure)—see Figure 4a inset for additional visualization. Also, expectedly, a drastic increase of Raman signal occurs: more than 4 orders of magnitude when the number of particles increases from 80 to 1300 (fill factor grows from 2.5% to ~40%). This is because of the increase in the number of “hot spots”. These results are qualitatively the same for arbitrary average radius of particles.

In Figure 4b, similarly, the magnitude of the PSD grows with the increase of the average radius of the particles. However, in this case the inflection point shifts to the low spatial frequency region, i.e., the correlation length increases. This is because the coverage of the surface is denser in the case of large particles (low period structure)—see Figure 4b inset for additional visualization. It is interesting to note that there is a noticeable shoulder in the high-frequency sloped region of PSD function for the ensemble with $\bar{R}$ = 16 nm. In our calculations, such shoulders appear in PSD functions for the ensembles with $\bar{R}$ >> σ. In these cases, sets of particles are relatively more “monodisperse” than ones with $\bar{R}$ ~ σ. Shoulders or even peaks in PSD functions are typical for the monodisperse ensembles [48]. One can see in Figure 4b, that Raman signal increases with the average radius. This is because the gaps between nanoparticles decrease. However, this growth is not as steep as it was in the case of varying number of particles: a little less than two orders for the radius increase from 4 to 16 nm. Note that in both cases (Figure 4a,b), the variation of fill factor is approximately the same: from 2.5% to ~40% (see the upper axis). This means that Raman enhancement is defined rather by the number of particles in the ensemble than by their sizes. Therefore, structures with the shorter correlation length are preferable for SERS. This statement is confirmed by the data presented in Figure 4c. In this figure we varied both the average radius and the number of particles, keeping the fill factor constant (40%). We observed that for the ensembles with a large number of smaller particles, the correlation length is shorter and Raman enhancement is higher than for structures with a smaller number of large particles (see inset in Figure 4c). Therefore, Raman enhancement is indeed primarily driven by the number of particles in the ensemble of hemispheroidal nanoparticles, and a shorter correlation length is preferable.

### 3.2. Experimental Studies

**Figure 5:**
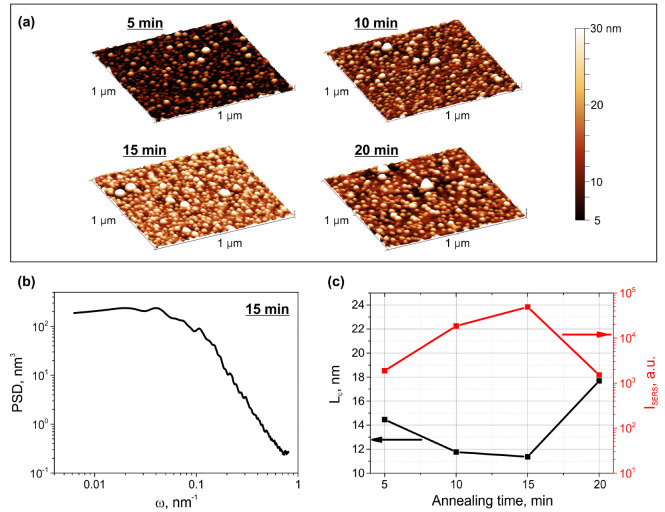
(**a**) AFM images of SNF formed at different annealing time (denoted). (**b**) PSD function of the AFM image of the 15-min sample. (**c**) The dependencies of integral intensity of 1200 cm^−1^ BPE Raman peak enhanced by SNFs (right axis, red curve) and SNFs’ correlation length (left axis, black curve) on the annealing time.

The AFM images of silver SNFs of different morphology formed on the glass surface at different annealing time are presented in Figure 5a. One can see that SNFs are ensembles of hemispherical/hemispheroidal nanoparticles, which are randomly distributed on the substrate surface. Thus, the surface topography of SNFs corresponds with the modeled one. To reveal the relation between SERS activity of different SNFs and their correlation lengths and compare experimental results with the numerical data, we analyzed 1D PSD functions of SNFs and performed SERS measurements using BPE molecules (see Section 2.3 for details). The correlation length was evaluated from PSD functions using Equation (3).

In Figure 5b we show an example PSD function of the AFM image of the 15-min sample. It noticeably well coincides with the modeled ones. In Figure 5c we present the dependencies of the integral intensity of 1200 cm^−1^ BPE Raman peak enhanced by SNFs and SNFs’ correlation length on the annealing time. Note, the 1200 cm^−1^ peak was used to quantify the SERS activity because of its insensitivity to BPE orientation on silver surface [49]. One can see, SERS signal increases, while the correlation length decreases, with the increase of the annealing time from 5 to 15 min. For SNF formed at 20 min of the annealing, SERS intensity drastically drops with the corresponding increase of the correlation length. Thus, SNF with the shortest correlation length, that is, SNF formed at 15 min of the annealing, provides the highest Raman enhancement. Indeed, the detailed analysis of the morphology of SNFs formed at different annealing time presented in our previous work [24] revealed that SNF formed at 15 min of the annealing contains the largest number of “hot spots” providing highest field enhancement. This result corresponds well with the results of the statistical and electrodynamics modeling performed in this study (see Figure 4a) and confirms that one can use the correlation length as the parameter defining the SERS-activity of ensembles of hemispheroidal metal nanoparticles.

**Figure 6:**
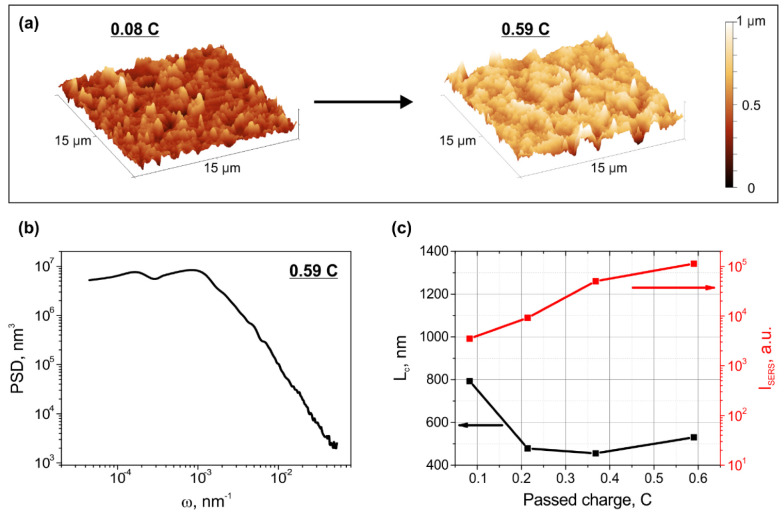
(**a**) Typical AFM images of the dendrites formed on the glass surface via electrolysis for passed charge 0.08 C (left) and passed charge 0.59 C (right). (**b**) PSD function of dendrites formed after passing 0.59 C. (**c**) The dependencies of integral intensity of 1200 cm^−1^ BPE Raman peak enhanced by dendrites (right axis, red curve) and dendrites’ correlation length (left axis, black curve) on the passed charge.

To verify the applicability of the correlation length for defining the SERS efficiency in the case of nanostructures different from nanoparticles, we analyzed 1D PSD functions and SERS-activity of silver dendrites formed in the glass via electrolysis with different passed charge (see Section 2.3 for details). The typical AFM images of the glass surface with the dendrites are presented in Figure 6a. From the images presented it is clear that the surface morphology varied depending on the passed charge: dendrite spikes become noticeably higher when passed charge increases from 0.08 C (left image) to 0.59 C (right image). In Figure 6b we present a PSD function of dendrites formed after passing 0.59 C. As discussed above this was obtained via superimposing of PSD functions based on AFM and optical profilometer. The PSD function is significantly shifted to lower frequencies comparing to one of the SNF (Figure 5b). This is because dendritic structures are significantly larger than the particles in the island film: microns vs. tens of nm. In Figure 6c we demonstrate the dependencies of the integral intensity of 1200 cm^−1^ BPE Raman peak enhanced by dendrites and dendrites’ correlation length on the passed charge.

In Figure 6c one can see the Raman enhancement provided by the dendrites increases with the increase of the passed charge, while the correlation length tends to decrease. This tendency is similar to ones observed experimentally and numerically for SNF. We should point out that the fractal nature of the dendrites, their nonuniform size and spatial distributions make it difficult to evaluate the average radius and the number of dendrites, describe their shape and form, and, therefore, perform electrodynamic modeling. For the complete description of the dendrites’ surface topography, only analysis of the second-order statistical function, PSD analysis, is applicable. Therefore, observed correlation between the correlation length evaluated from PSD functions and Raman enhancement allows simple identification and choosing of a structure capable of providing maximal enhancement of Raman signal. This confirms that one can use the correlation length as a universal parameter defining the SERS efficiency of nanostructures placed on the substrate surface.

## 4. Conclusions

Finally, we have modelled a set of structures differing in size and number of randomly distributed hemispheroidal metal nanoparticles placed on glass substrate and evaluated awaited magnitude of Raman signal enhanced by each of the structures. In spite of arbitrary scale of the calculated Raman signal, these allowed us to compare the signals enhanced by different structures. Performed PSD analysis of the structures has established a relation between their lateral correlation length and enhanced Raman signal. This relation was verified in experiments with silver nanoisland films, which confirmed that shortest lateral correlation length determined using PSD function corresponds to the structure providing the highest Raman signal. Application of this approach to experimental data on Raman signal enhanced by silver dendrites, which topography essentially differs from the nanoisland films, has shown that the correspondence of shorter lateral correlation length to higher Raman signal is valid in this case also. Thus, it is demonstrated that simple analysis of PSD function, which can be obtained using standard software for processing AFM data on surface roughness, allows comparing SERS capability of similar structures and choosing ones providing the highest Raman signal.
